# The effect of silica nanoparticles on the mechanical properties of fiber-reinforced composite resins

**DOI:** 10.15171/joddd.2016.018

**Published:** 2016-06-15

**Authors:** Mohammad Bagher Rezvani, Mohammad Atai, Faeze Hamze, Reihane Hajrezai

**Affiliations:** ^1^Assistant Professor, Department of Operative Dentistry, Shahed Dental School, Shahed University, Tehran, Iran; ^2^Dental Research Center, Shahed Dental School, Shahed University, Tehran, Iran; ^3^Iran Polymer and Petrochemical Institute (IPPI), Tehran, Iran; ^4^Assistant Professor, Department of Operative Dentistry, Faculty of Dentistry, Kerman University of Medical Sciences, Kerman, Iran

**Keywords:** Fiber-reinforced composite resins, flexural strength, nanoparticles, silicon dioxide

## Abstract

***Background.*** Nanotechnology has introduced many nanoparticles in recent years, which can be incorporated for mechanical improvement of dental materials. However, the existing data are widely sparse. This study investigated the reinforcing effect of silica nanoparticles when incorporated into the matrix phase of an experimental dental fiber-reinforced compositeresin (FRC) through evaluation of its flexural properties.

***Methods.*** In this experimental study FRC samples were divided into two main groups (containing two or three bundles),either of whic consisted of five subgroups with 0, 0.2, 0.5, 2 and 5 wt% of silica nanoparticles in the matrix resin (n=10 in each subgroup); a commercial FRC (Angelus, Brazil) was used as the control group (n=10). Three-point bending test was performed to evaluate the flexural strength and modulus. Thereafter, the microstructure of the fractured samples was evalu-ated using scanning electron microscopy (SEM). The results were analyzed with one-way ANOVA and HSD Tukey tests (α = 0.05).

***Results.*** The results revealed that the silica nanoparticles had a significant and positive effect on the flexural strength and modulus of FRCs (P<0.05), with no significant differences from 0.2 to 5 wt% of nanoparticles (P > 0.05) in either group with two or three bundles of fibers.

***Conclusion.*** Incorporating silica nanoparticles into the FRC resin phase resulted in improved flexural strength and modulus of the final product.

## Introduction

Fiber-reinforced composite resins (FRC) are increasingly used in modern dentistry as a substitute for metal frameworks in crowns, bridges, denture bases, orthodontic appliances and periodontal splints.^1,2^ In general, fiber reinforcement provides superior performance and enhanced mechanical properties in composite substrate, especially in relation to tension and flexure.^2^ Among various types of fibers, glass fibers seem to be the most favorable in dental applications because of their high esthetic properties, chemical resistance and relatively low cost.^2^ Furthermore, they have a good adhesion capacity to mono- and dimethacrylates after silanization.^3^

However, the application of FRCs has been restricted to short spans because the most important drawback of the FRCs is still their flexural strength that limits their application in long-span prostheses.^4^ Therefore, many researches were designed to improve the flexural strength of FRCs, leading to a wide range of data.^1,2^ An important methodology aiming to improve the performance of FRCs was incorporating different fillers into composite materials. The fillers had a considerable effect on the mechanical behavior that is absolutely dependent on the shape and size of the fillers.^5-7^

Nanotechnology, however, introduced many nanoparticles in recent years which are widely used for mechanical improvement of dental materials.^8,9^ It has been frequently documented that different nanoparticles significantly enhance the flexural strength of dental resin matrix.^10^ Actually, nanoparticles were more effective for mechanical reinforcement of resin matrix compared to macroparticles.^11^Among these various nanoparticles, silica is desirable in dental resins because it has high strength and superior esthetic features.^12^

Accordingly, some previous researchers incorporated nanoparticles into FRC structures^13^ and reported that un-modified nanoparticles did not have any significant effect on the mechanical properties while resin-grafted nanoparticles significantly enhanced the flexural strength of FRCs.^13^ In contrast, some other investigators revealed that incorporating different simple un-modified nanoparticles into FRCs would noticeably enhance the mechanical properties of FRC.^14,15^ However, the application of nanoparticles for reinforcement of FRCs is still under investigation and the dental literature lacks adequate information while the existing documentation shows a very sparse range of data. Therefore, this study was designed to investigate the reinforcing effect of silica nanoparticles when incorporated into the matrix resin of the experimental dental FRC through the evaluation of the flexural properties of the composite resins. The effect of fiber content was also studied.

## Methods

This study did not involve the use of any animals or human data or tissues, and thus, an ethics approval was not required.

2,2-Bis-(2-hydroxy-3-methacryloxypropoxy) pheny propane (Bis-GMA) and triethyleneglycoldimethacrylate (TEGDMA) were purchased from Evonic (Germany). Camphorquinone (CQ) and N,N′-dimethyl aminoethyl methacrylate (DMAEMA) were obtained from Fluka (Germany). Glass fibers (E-Glass, tex=2400 g/1000 m) were obtained from Kamelyaf (Turkey), amorphous silica nanoparticles with a primary particle size of 10 nm (HDK, N-20) were obtained from Wacker (Germany). A commercially available dental FRC was purchased from Angelus (Brazil) and tested as the control group.

### Resin preparation

A mixture of 60 wt% of Bis-GMA and 40 wt% of TEGDMA was prepared as the matrix phase. The nanoparticles were then added to the matrix in different percentages (0, 0.2, 0.5, 2, and 5 wt%) at sub-ambient light environment. In order to prevent agglomeration of the nanoparticles, the mixture was shaken for 24 h and sonicated for 5 min (SONOPlus, BANDELIN, Germany) at the sub-ambient light. Then, 0.5 wt% of camphorquinone and 0.5 wt% of *N,N*-dimethylaminoethyl methacrylate, as light-curing initiator system, were dissolved in the matrix and the whole mixture was shaken again for 24h at the sub-ambient light.

### Sample preparation

In this experimental study FRC samples were divided into two main groups (containing two or three bundles), either consisting of five subgroups with 0, 0.2, 0.5, 2 and 5 wt% of nano-SiO_2_ in the resin phase (n=10 in each subgroup), while the commercially available FRC (Angelus) was used as the control group (n=10).

Each glass fiber bundle was impregnated with the prepared resin matrix prior to inserting into the rectangular 25×2×2-mm stainless steel mold which was placed on a glass slide. Two or three fibers (according to the grouping) were inserted in each mold and the remaining space of the mold was filled with the prepared experimental resin related to each sub-group. However, in the control group (Angelus FRC) the mold was filled with seven bundles (because this type of FRC is marketed as fibers embedded in composite resin and seven bundles of this composite containing FRC was needed to fill the whole mold). Then, the mold was covered by another glass slide and the specimens were cured from both top and bottom sides by a light-curing unit (Optilux 501, Kerr, USA, with an intensity of 600 mW/cm^2^) for 40 s in each spot using an overlapping regimen. The specimens were removed from the mold and stored in deionized water at 37°C for one week. Subsequently, the samples underwent 500 thermal cycles between 5 and 55°C prior to the test (Thermocycler, VafaiIndusterial LTD, Iran). Both surfaces of all the specimens were polished using sand paper in a moist environment.

### Flexural strength and modulus

A three-point bending test was performed using a universal testing machine (Z20, ZwickRoell, Germany) at a cross-head speed of 0.5 mm min.^1^ The flexural strength (FS) in MPa was calculated as:^10^

FS =3PL/2bd^2^

where *P* stands for load at fracture (N), *L* is the span length (20 mm),^10^ and *b* and *d* are, respectively, the width and thickness of the specimens in millimeter (both of them are 2 mm for all samples).^10^ The elastic modulus was also determined from the slope of the initial linear region of stress–strain curve.

### SEM

Microstructure of the fractured surfaces obtained from flexural strength test was analyzed using SEM (TESCAN, VEGAII, XMU, Czech Republic). The samples were mounted on the aluminum stub using carbon-coated double sided adhesive tape and then coated with gold using a sputter coater.

### Statistical analysis

Data were analyzed using one-way ANOVA and HSD Tukey tests (α = 0.05).

## Results

### Flexural strength and modulus

The mean flexural strength and modulus of the study groups are presented in Table 1 and Table 2.

**Table 1:** The flexural strength (MPa) ± SD of the experimental samples containing 0 to 5 wt% of SiO_2_ nanoparticles and the control group (Angelus)

**Groups**	**0 wt%**	**0.2 wt%**	**0.5 wt%**	**2 wt%**	**5 wt%**	**Angelus**
**2 bundle group**	64.2 ± 11.28^a^	90 ± 17.34^b^	94.5 ± 13.85^b^	97.1 ± 13.95^b^	100.2±18.41^b^	40.4 ± 19.28^c^
**3 bundle group**	87.4 ± 14.09^d^	115.3±17.53^e^	113.1±13.68^e^	114.9±23.82^e^	125±23.84^e^	

Same superscript letter within the value represents homogenous subset (α=0.05).

**Table 2:** The flexural modulus (GPa) ± SD of the experimental samples containing 0 to 5 wt% of SiO_2_ nanoparticles and the control group (Angelus)

**Groups**	**0 wt%**	**0.2 wt%**	**0.5 wt%**	**2 wt%**	**5 wt%**	**Angelus**
**2 bundle group**	11.7 ± 4.52^a^	15.5 ± 4.97^b^	16.1 ± 4.22^b^	15.9 ± 4.88^b^	16 ± 3.16^b^	7.4 ± 1.83^c^
**3 bundle group**	14.5 ± 4.79^d^	23.5 ± 8.57^e^	23.9 ± 5.38^e^	23.8 ± 8.23^e^	21.8 ± 4.93^e^	

Same superscript letter within the value represents homogenous subset (α=0.05).

As can be seen, the control group (Angelus) has the lowest flexural strength and modulus which are significantly lower than those of the experimental groups (P-values are demonstrated in Tables 3 and 4).

**Table 3:** The matrix P-values between different treatments in flexural strength of two-bundle (below the diagonal) and three-bundle (above the diagonal) groups

**Treatments**	**Angelus**	**0%**	**0.2%**	**0.5%**	**2%**	**5%**
**Angelus**	**0**	0.000^*^	0.000^*^	0.000^*^	0.000^*^	0.000^*^
**0%**	0.018^*^	**0**	0.023^*^	0.044^*^	0.026^*^	0.001^*^
**0.2%**	0.000^*^	0.008^*^	**0**	1.000	1.000	0.866
**0.5%**	0.000^*^	0.001^*^	0.988	**0**	1.000	0.733
**2%**	0.000^*^	0.000^*^	0.917	0.999	**0**	0.845
**5%**	0.000^*^	0.000^*^	0.709	0.966	0.998	**0**

P-values are significant at the 0.05 level.

**Table 4:** The matrix P-values between different treatments in flexural modulus of two-bundles (below the diagonal) and three-bundle (above the diagonal) groups

**Treatments**	**Angelus**	**0%**	**0.2%**	**0.5%**	**2%**	**5%**
**Angelus**	**0**	0.012^*^	0.000^*^	0.000^*^	0.000^*^	0.000^*^
**0%**	0.022^*^	**0**	0.002^*^	0.001^*^	0.001^*^	0.010^*^
**0.2%**	0.000^*^	0.042^*^	**0**	0.883	0.912	0.534
**0.5%**	0.000^*^	0.020^*^	0.744	**0**	0.971	0.443
**2%**	0.000^*^	0.026^*^	0.828	0.913	**0**	0.464
**5%**	0.000^*^	0.022^*^	0.786	0.957	0.957	**0**

^*^ P-values are significant at the 0.05 level.

However, the flexural strength and modulus increased with an increase in nanoparticle content. In fact, for either of the main groups (containing two or three fibers), 0 wt% nanoparticle samples exhibited significantly lower flexural strength and modulus compared to the 5 wt% samples. There was, however, no statistically significant difference among nanoparticle-containing samples. It means that incorporation of nanosilica particles into the resin matrix had a significant effect on the flexural properties of FRCs even when the nanoparticle content was as low as 1 wt%.

### SEM analysis

**Figure 1.**
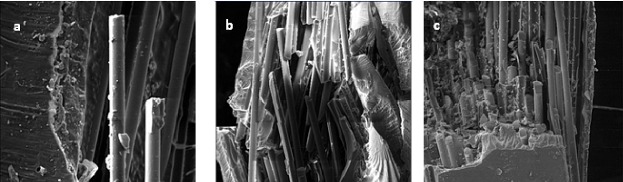
The SEM micrograph of fractured samples containing 0 wt% (a) and 5 wt% (b) SiO2 nanoparticles.

As shown in  Figure 1, by increasing the nanoparticle content in the resin matrix, the fibers are coated with greater amounts of the filler. Accordingly, at the fractured surface of 0 wt% sample the fibers are completely delaminated from the resin matrix while in the 5 wt% sample there is good adhesion between the fibers and the resin matrix, indicating that incorporating nanosilica particles into resin matrix improved FRC properties because an effective bond between the resin matrix and fibers is always a desirable and important phenomenon in FRCs.

## Discussion

The results showed that incorporation of silica nanoparticles up to 0.5 wt% had a significant and positive effect on the flexural strength and modulus of FRC while there were no significant differences from 0.2 to 5 wt% nanoparticles in either group with two or three fibers.

This finding is consistent with many previous researchers who reported mechanical improvement by incorporating various nanoparticles into composite resins.^16-19^ In contrast, a few investigators have claimed that no mechanical enhancement of resin matrix was achieved by nanoparticles.^3,20^ Chisholm et al compared the effects of different fillers on the flexural strength of resin matrix and reported that the nanoparticles were considerably more effective compared to macro-fillers, which might be attributed to their higher surface energy.^11^

Moreover, Sfondirini et al^15^ investigated nano-filled FRCs, and similar to our results, reported that nanofilled FRCs showed higher load values compared to conventional forms. In contrast, in another survey, Mortazavi et al^13^ showed that when simple nanoclay particles were mixed with the resin matrix in FRCs, the flexural strength did not improve significantly. However, when the nanoclay particles were grafted to poly(methyle methacrylate) the flexural strength of FRCs increased significantly.^13^

Unidirectional FRCs have high flexural strength because the reinforcing fibers prevent crack propagation at the microscopic substructure.^21^ It has been shown that the flexural strength and modulus of some commercial FRCs are seven times higher than the same resin with particulate fillers.^2^ Among various commercially available FRCs, we selected the Angelus FRC (considered as the control group) because its composition is very similar to our experimental samples. According to the manufacturer’s data, Angelus FRC consists of E glass fibers while they are highly packed by silicon dioxide. Moreover, both the E glass fibers and silica particles were incorporated in this study due to their high esthetic performance, mechanical properties and their popularity in dental materials compared to the other similar compounds.^12^ The results of the current survey show that although the flexural strength of the two-bundle group increased with an increase in nanoparticle content, the three-bundle group exhibited a different trend (Tables 1 and 2).

Accordingly, in the three-bundle group the flexural strength of 0.5 wt% group was less than 0.2 wt% group and the flexural modulus of 5 wt% group was lower than 0.5 wt% samples. This finding could be explained by the fact that the high percentage of nanoparticles would increase the viscosity of resin phase and subsequently, it could not impregnate the glass fibers properly.^13^ Therefore, it could be claimed that in the three-bundle group which had a much more surface-to-volume fraction of fibers to matrix, the more viscous resin would lead to improper bond of fiber to resin, resulting in lower mechanical strength.

On the other hand, it has been documented that if the resin phase becomes overloaded with nanoparticles, internal porosities and mass irregularities would form in the resin phase that would reduce its strength.^22,23^ Moreover, in previous investigations it has been argued that for mechanical enhancement of the resin matrix the amount of incorporated nanoparticles has a threshold above which the strength would decrease.^6,16^ Therefore it could be concluded that incorporating nanosilica particles would be beneficial in FRC resin matrix up to around 0.5 wt%.

In our experiment both the flexural strength and modulus of the three-bundle group was higher than the two-bundle group. This finding was quite predictable because it has been frequently documented that higher fiber content leads to higher flexural strength parallel to the fibers' orientation.^24^

One of the most interesting outcomes of the current research was that although the nanoparticles were not silanized, the SEM micrographs displayed that as the nanoparticle weight fraction increased, the fibers exhibited better adhesion to the resin matrix. As it is demonstrated in Table 2, the 0 wt% group showed complete delamination of fibers from the resin matrix while the fractured fibers in 5 wt% group were still impregnated in the resin matrix. This could be related to the high surface energy of nanoparticles.^12^ Accordingly, it could be claimed that more SiO_2_ nanoparticles guarantee a more effective bond between fibers and the resin matrix. Since prior documentations revealed that the rigidity and strength of FRC is significantly influenced by the quality of impregnation by resin matrix,^25^ a higher content of SiO_2_ nanoparticles would be advantageous in this aspect. However, it should be emphasized that the amount of nanoparticles has a threshold beyond which no further mechanical enhancement would be achieved due to possible formation of defects and flaws.^26,27^

## Conclusion

Incorporating SiO_2_ nanoparticles into FRC resin phase not only had a significant effect on its mechanical behavior but also led to a more proper impregnation of fibers.

## Acknowledgments

The authors would like to thank the Dental Research Center of Shahed Dental School (Tehran, Iran) for financial support of the study.

## Authors’ contributions

MBR and MA were responsible for the design and concept of the study as well as revising the prepared manuscript. FH analyzed the data, carried out the literature search, and drafted the manuscript. RH performed the experiments. All the authors have read and approved the final manuscript.

## Funding

This research has been financially supported by Shahed Dental School.

## Competing interests

The authors declare no competing interests with regards to the authorship and/or publication of this article.

## Ethics approval

Not applicable.
